# The Key Aspects of Neonatal and Infant Neurological Examination: The Ballard Score, the Infant’s Head with Hydrocephalus and Assessment in a Clinical Setting

**DOI:** 10.21315/mjms2023.30.4.16

**Published:** 2023-08-24

**Authors:** Zaitun Zakaria, Hans Van Rostenberghe, Noraida Ramli, Mohamad Syahrain Suhaimi, Siti Nur Haidar Hazlan, Jafri Malin Abdullah

**Affiliations:** 1 Department of Neurosciences, School of Medical Sciences, Universiti Sains Malaysia, Kelantan, Malaysia; 2 Department of Neurosciences, Hospital Universiti Sains Malaysia, Universiti Sains Malaysia, Kelantan, Malaysia; 3 Brain and Behaviour Cluster, School of Medical Sciences, Universiti Sains Malaysia, Kelantan, Malaysia; 4 Department of Paediatrics, School of Medical Sciences, Universiti Sains Malaysia, Kelantan, Malaysia; 5 Department of Paediatrics, Hospital Universiti Sains Malaysia, Universiti Sains Malaysia, Kelantan, Malaysia

**Keywords:** Ballard score, hydrocephalus, infant, maturity, neonates, neurology

## Abstract

The physical examination of the newborn is essential in diagnosing neurological or neurosurgical conditions in the newborn. This article focuses on three clinical assessments of newborns and infants that are especially important if neurological problems are suspected: The Ballard score, the examination of the head in a baby with (suspected) hydrocephalus, and the neurological and developmental evaluation of an infant in an ambulatory setting. A textual description and a link to a video describe each assessment.

## Introduction

Neonates and infants could not communicate their symptoms to healthcare professionals, rendering a close observation and good physical examination essential in neonatal care. The neonatal brain is very fragile and perinatal events such as difficult delivery or prematurity pose a significant risk to the developing brain. Hypoxic ischaemic brain injury in both term and preterm neonates and intraventricular hemorrhages, mainly in preterm neonates, are common complications in the neonatal intensive care unit (NICU). Hydrocephalus, either congenital or as a consequence of the complications mentioned earlier, is a serious condition requiring adequate assessment of the baby.

Most high-risk neonates admitted to the NICU for a prolonged time require close follow-up as outpatients. Again, a thorough physical examination by an experienced doctor is vital to detect early neurological problems or developmental delays. This article focuses on three important areas of the neurological assessment of neonates: i) maturational assessment of gestational age using the Ballard score, ii) neurological examination of the infant’s head with suspected hydrocephalus and iii) neurological examination in a clinical setting.

### Maturational Assessment of Gestational Age Using the Ballard Score

Gestational age describes the time elapsed between the first day of the last normal menstrual period and the delivery of the baby (1). It is crucial for healthcare workers caring for sick neonates to know the gestational age as accurately as possible. The most reliable determination of gestational age is through the mother’s menstrual history when the mother is certain about the first day of the last menstrual period. Early prenatal ultrasonography is considered the second most reliable method to assess the gestational age of a fetus. It relies upon the proportion of foetal growth rates, such as crown-rump length measured in the first trimester and a biparietal diameter measured in the second trimester (2).

**Figure 1:**
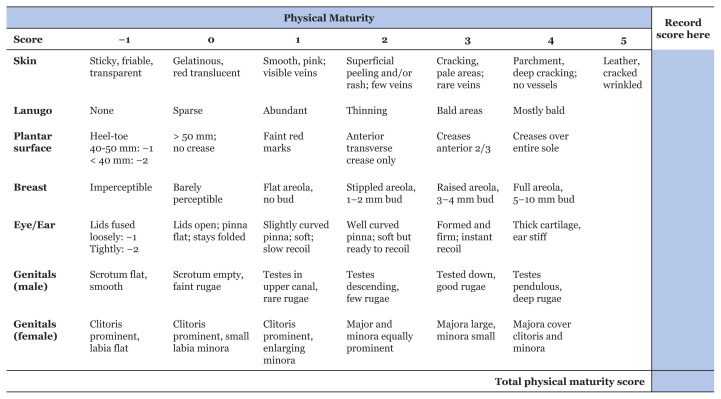
The physical maturity assessment for Ballard score. Each assessment gets a score between −1 and maximum 5

**Figure 2:**
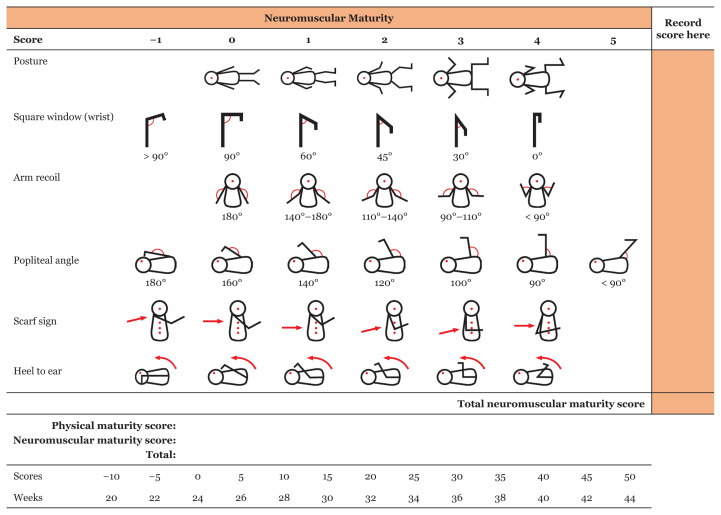
The neuromuscular maturity assessment for Ballard score and the correlation between the score and the gestational age

However, certainty about the first day of the last menses and early antenatal ultrasound is often unavailable, especially in low- and middle-income countries. That is when one can fully appreciate the usefulness of a scoring system, such as the new Ballard maturation score (3, 4). The validity of this examination may extend until at least day 7 of life (5). In some cases, the Ballard score indicates a gestational age that is about 4 weeks more than the gestational based on the first day of the last menstrual period. This may mean that there has been decidual bleeding at about 4 weeks of gestation, which may have been misinterpreted by the mom as menses. If a detailed history shows that the duration or intensity of the last menses was less than usual, the Ballard score may indicate the true gestational age. The maturational assessment is divided into physical (Figure 1) and neuromuscular maturity (Figure 2). Each category involves six assessments to bring a score of 10–50. The assessment is demonstrated in Video 1 (https://youtu.be/xlcWbH3Ym_g) and explained in detail as follows.

#### Physical Maturity

- The first assessment for physical maturity is the skin. The maturation involves the development of its intrinsic structures concurrent with the gradual loss of its protective coating, the vernix caseosa. As foetal maturation progresses, the skin becomes thickened, wrinkled, and starts peeling. At term and post-term, the skin started to dry, causing further peeling and cracking with a leathery appearance.
- Lanugo is the fine, soft, and unpigmented hair resembling ‘peach fuzz’ covering the body of the fetus. Premature infants of 24 weeks–32 weeks gestation frequently display this fine coat of lanugo hair. The bald area starts to occur at the lumbosacral area and term babies will be devoid of lanugo.
- The plantar surface of the foot is scored, mainly based on its creases. At 28 weeks gestation, the sole appears smooth. The creases start to appear at the ball or anterior one-third of the foot and accelerate with increasing maturity. In a very immature infant, the heel-toe distance should be measured.
- The breast areola and bud should be felt and measured, with a value in millimeters. At 28 weeks gestation, the breast tissue is absent and the areola is barely visible. By 32 weeks gestation, the areola is stippled with a raised bud that can be measured.
- The eye/ear involves looking at the changes in configuration/curving of the pinna, thickness of cartilage, and state of development of eyelids as maturity increases. For example, at 26 weeks gestation, the ear has less cartilage and is pliable; hence, folding of the pinna will cause a slow release or it will remain folded. However, in term babies, the ear is firm with a well-formed margin. The pinna will instantly recoil.
- Genitals (male) involve an examination of the scrotal area. At 28 weeks gestation, the testicles are still high in the scrotum. The left testicle precedes the right, descends and enters the scrotum during 32 weeks gestation. The scrotal rugae can be appreciated at this stage. The scrotal skin becomes thicker and pigmented, and at 36 weeks gestation, both testicles are well-descended and palpable.
- Genitals (female) involve an examination of the clitoris, labia minora and majora. The neonate is positioned supine with the hips abducted to approximately 45°. At 28 weeks gestation, the labia are flat and the clitoris is prominent. Increasing fat deposit in labia majora occurs at 32 weeks gestation, and during this stage, both clitoris and labia minora recede. By 36 weeks gestation, labia majora will envelop and cover the labia minora and clitoris.

#### Neuromuscular Maturity

- Posture reflects body muscle tone at rest. The maturation starts in a centripetal direction, and the lower extremity is ahead of the upper extremity.
- The square window test determines the wrist flexibility and/or resistance to extensor stretch. The examiner straightens the fingers and applies pressure to the dorsum of the hand. The angle when the neonate’s wrist is flexed progresses from 90° to 0° with advancing gestational age. The 45° angle is consistent with a gestational age of 30–32 weeks.
- The arm recoil maneuver looks at the passive flexor tone of the biceps muscle by measuring the recoil after a brief arm extension. The examiner should place one hand under the neonate’s elbow for support. Arm recoil measures from 180° to < 90°. Score 4 should be given when the arm recoils and the fist touch the face.
- The popliteal angle is measured when the infant’s thigh is flexed at the hip and placed at the abdomen. The examiner should support the thigh from the side and avoid excessive pressure on the flexor being tested. Then, the examiner holds the foot and extends the leg below the knee until resistance is felt. The angle progresses from 180° to < 90° with increasing gestational age.
- The scarf sign is performed to test the passive tone of the shoulder girdle. The head should stay in the midline, and the examiner takes the infant’s hand and places it around the neck as far as possible while feeling the resistance of the muscle. The score is based on the point of the elbow on the chest when the resistance occurs.
- The heel-to-ear test measures the resistance of the flexor muscles at the hip. The hip girdle is stabilised and the hip is flexed so that it rests on the sheets alongside the neonate’s trunk. Then, the leg is drawn towards the ear to see the resistance to the maneuver.

Again, each assessment is given a score between −1 and a maximum of 5. In the end, all scores are added up to determine the corresponding gestational age.

### Neurological Exam of the Head of an Infant with Suspected Hydrocephalus

The second video (Video 2: https://youtu.be/YADjepw_5mk) shows the clinical evaluation of the head of a child that was born with or who was developing signs of hydrocephalus within the first month of life. Details on the process of the physical examination are given in the text below.

Understanding the signs and symptoms of hydrocephalus helps address further treatment plans (6). The clinical history is commonly related to structural brain abnormalities, premature birth, birth trauma, intracranial or intraventricular haemorrhage and central nervous system (CNS) infection. The parents or the primary team would notice symptoms of irritability in cases with increasing intracranial pressure. Poor sucking on the breast or refusal to suck are important warning signs. The infant may not stay awake during feeding or appear not hungry. Nausea and vomiting may occur either spontaneously or induced when being fed via a bottle or nasogastric tube. The neonatologist would find it difficult to wean the ventilatory support in an intubated baby due to apnea. The new onset of a partial and generalised seizure or increased seizure frequency is attributed to alterations in cerebral blood flow and cortical irritability (7). The sound of a high shrill cry may also be heard. The presence or absence of the above symptoms should be documented for every child with suspected hydrocephalus.

The environment influences the baby’s behavior during the assessment. Although the best time is when the baby is quiet and awake, with adequate light and environment temperature at 28 ± 2 °C, sometimes these are not feasible when performed on a sick baby or in a busy emergency room. The sequence of neurological examination techniques, i.e. inspection, palpation, percussion and auscultation, are a matter of personal preference and must be tailored to the exam. A close inspection may reveal dilated or prominent scalp veins secondary to the reversal of flow from intracerebral sinuses (8).

One needs to be observant of the baby’s head, which may seem disproportionately larger or bigger, with an unusually prominent forehead (frontal bossing) and lateral bulging of the parietal bones. The shape of the head may vary based on the cause of hydrocephalus and the underlying condition of the skull bones. A disproportionately large forehead is common in aqueductal stenosis. A combination of other features, such as midface retrusion or hypoplasia, hyper- or hypotelorism, softening of the skull (craniotabes), and/or skeletal abnormalities, may suggest an underlying genetic syndrome (9). Occipital prominence is appreciable in a baby with a Dandy-Walker malformation (10). Frontal and occipital prominence may indicate sagittal synostosis (scaphocephaly), while a widened transverse diameter of the skull (broad and short) is suggestive of bicoronal synostosis (brachycephaly) (11).

#### Palpation

Palpation of the neonatal head starts with general palpation to feel for bruising, abrasion or swelling, such as caput succedaneum, cephalohematoma, or subaponeurotic haemorrhage (SAH). Clinical differentiation of these three scalp-swelling types is essential since each has different clinical implications. Cephalohematoma does not cross suture lines but fluctuates (boggy) on palpation. SAH is also fluctuant on palpation but crosses the suture lines. Meanwhile, caput succedaneum crosses the suture lines but is not fluctuant.

Palpation along the suture line allows the doctor to appreciate widely spaced or split sutures. The sutures, namely the sagittal, coronal, lambdoid and metopic are separated by connective tissue junctions to allow growth along the edges of the two bones and permit adequate space for the growth of the brain during the development of the infant into childhood (12). The palpable sutures can be mobile, split or separated up to 1 cm. The sutures may feel overriding (common for the lambdoidal sutures) (13). Craniosynostosis involves abnormal mineralisation of the sutures and the premature fusion of one or more bones of the cranial vaults (14). It should be suspected in abnormal cranial shapes (see above) in combination with sutures that feel ridged rather than overriding (13).

#### Fontanel

Four palpable fontanels are noted in a quiet infant, i.e. anterior, posterior, sphenoidal and mastoid. They should be soft and flat. Upon uncertainty of whether the fontanel is flat or bulging, the infant should be placed in an upright, sitting position for further palpation. Palpation of the anterior fontanel feels like the shape of a diamond, with an average size of 2.1 cm. The posterior fontanel feels like a triangle and is small enough to admit the tip of a finger. Variations in the size and closure time of the fontanels depend on gender and ethnicity (15–17). However, one would expect that the first fontanel to close is the posterior fontanel, generally at 2 to 3 months after birth, followed by the sphenoidal fontanel at around 6 months after birth, mastoid fontanel around 6 to 18 months after birth, and the anterior fontanel around 6 to 24 months after birth (median time of closure is 13.8 months) (17, 18).

Upon the presence of hydrocephalus, the examiner may feel for a full, bulging and non-pulsating anterior fontanel. The fontanel tends to bulge when the infant cries. However, due to the easy expansion of the skull, the fontanel may not bulge or feel tense. The only sign may be an increased size of the fontanel.

#### Occipitofrontal Circumference

The head circumference or occipital frontal circumference (OFC) should be measured using a flexible non-stretchable measuring tape. The following should be considered to improve the reading and prevent discrepancies in measurements:

- The infant and examiner are in a comfortable position.
- Any hair decorations that interfere with the measurement must be excluded.
- The tape must be snug over the most prominent part on the back of the head (occiput) and just above the eyebrows (supraorbital ridges).
- Two measures are to be taken, and the tape has to be repositioned each time. The largest value is recorded upon 0.2 cm differences in measurement. If the measurements exceed the tolerance value, a third measurement is taken, and the average of the two closest measures is recorded.

According to the standard growth curves for head circumference by the World Health Organization (WHO), microcephaly at term is described as the OFC < 32.1 cm for boys and < 31.7 cm for girls, whereas macrocephaly is when the OFC > 36.9 cm for boys and > 36.1 cm for girls (19). The Malaysian term newborns have a smaller OFC, with 30.5 cm for boys and 30.0 cm for girls for the 3rd percentile, and 35.5 cm for boys and 35.0 cm for girls for the 97th percentile. The average (at the 50th percentile) OFC is 33.0 cm for boys and 32.5 cm for girls (20). A head circumference of more than 2 standard deviations (SD) above the mean for sex and gestational age is known as macrocephaly. However, considering that OFC of 2–3 SD is found in 2% to 3% of the normal population, macrocephaly becomes ‘clinically relevant’ above 3 SD (9).

#### Transillumination

An abnormal fluid-filled intracranial cavity can be transilluminated using a strong torchlight. The transillumination test should be performed in total darkness and starts against the infant’s forehead (midline near the hairline or at the anterior fontanel), occiput to forehead, and side to side. A faint glowing subcutaneous halo around the circumference of the flashlight is normal. Larger, irregular and brighter areas may indicate the presence of an abnormality, especially in a thin skull. When the transillumination is seen from the side opposite of a flashlight, an appearance of total glowing is usually suggestive of hydranencephaly. An asymmetrical cranial transillumination of one side may reveal the abnormal area of enlarging cystic or cerebrospinal fluid spaces (21).

#### Percussion and Auscultation

On percussion, a ‘cracked pot’ resonance sound (first described by William Macewen in 1893) can be heard when percussing near the junction of the frontal, temporal and parietal bones. This happens as the bone becomes thin due to increased distance between the sutures of the bones and the ready vibration of the bone when the content is more fluid (22). Next, trans-fontanel auscultation should be performed to look for bruits, such as in the case of vein of Galen malformation (23).

#### Eye Examination and Other Parts

After completing the examination around the scalp, the examiner should proceed with checking the eyes for position and pupillary sizes. Common abnormal eye positions in infants with hydrocephalus are either deviating downwards (setting sun sign) due to pressure on the suprapineal recess or deviating medially (convergent squint) due to weakness on the lateral rectus muscle. Sometimes, the observations might not be obvious and require the oculocephalic or vestibulo-ocular reflexes, whereby the baby’s head is turned from side to side or up and down (24, 25). The normal finding is when the eye deviates in the opposite direction to the head movement.

Next, the reaction of the pupils to light should be tested for both direct and indirect reflexes. Even when the eyes are closed, the blink reflex to light can still be tested unless the baby is crying, when the eyes are already held maximally closed. General depression from CNS, such as anoxia, sedation from anesthesia or a comatose baby, causes a diminished reflex. A fundoscopic exam is also important to search for papilledema. The examination of an infant with (suspected) hydrocephalus should be completed by examination of the other cranial nerves, a complete neurological assessment and examination of the body of the child for the presence of deformations and/or malformations, determination of the tone of the baby and testing of reflexes.

### Neurological Exam in a Clinical Setting

The neurological examination of an infant should be tailored to their developmental level and cooperation. The examiner must acquire knowledge and appreciate the normal and abnormal findings. Sometimes, the neurological exam is performed with the infant on the parent’s lap to overcome shyness and stranger anxiety. Serial examinations may be necessary to establish accurate findings or diagnoses. In a busy clinic, a gross examination is performed to detect an abnormal pattern of neurological deficits. Once detected, further thorough assessment and referral to specific services are required. Children born preterm are expected to have developmental milestones and anthropometric measurements according to their corrected gestational age. The corrected gestational age is obtained by subtracting the number of weeks they were born before 40 weeks from their chronological age. Meanwhile, the chronological age is used only for immunisation and feeding practice for preterm babies.

At 1 year old of age, most of the gross motor milestones have appeared, and the concerned neurological findings such as hypo- or hypertonia, hypo- or hyperreflexia, and pathological and primitive reflexes observed in earlier testing should no longer be present to a significant degree (26). This section provides the general idea of neurological examinations that can be performed in a clinical setting: i) observation for the level of alertness, ii) examination of the head and spine, iii) cranial nerve function, iv) tone and muscle strengths, v) deep tendon reflexes, vi) primitive reflexes and vii) sensory examination.

#### Observation for the Level of Alertness

The ambience of the environment is vital during an examination of an infant. It is best performed in a warm room and during the infant’s quiet alert state, which most frequently occurs between feedings. The infant should lie down unclothed and in unconfined boundaries. This position enables accurate observation of facial and limb positions during the resting posture and spontaneous movements of the head and limb. The level of alertness can be viewed in Table 1. A normal young infant will have a normal physiological behavior of loud and lusty crying. A weak cry may indicate a depressed, lethargic, or dehydrated infant. An irritable and having meningitis infant usually has a high-pitched cry (27).

## Examination for syndromic features

Below are some of the less rare syndromic features to be looked at on a routine basis, without any intention to give a complete list of syndromic features.

### Cranium

The shape and size of the infant’s skull require careful inspection and palpation of the sutures and fontanel, followed by measurement of the OFC (please see the previous section).

### Facial features

The facial features involve observation and appreciation of the relationships of the facial components; ears, eyes, nose, mouth and mandible. An unusual facial characteristic may alert the examiner of certain syndromes or neurological injuries. For example, a pale-pink macular lesion (port-wine stain) over the face may signify Sturge-Weber syndrome (28). Facial movements during crying or even cooing are assessed for asymmetry. An injury to the seventh cranial nerve may occur because of birth trauma: commonly the mandibular branch of the facial nerve causing downward movement of the corner of the mouth on the normal side (drooping mouth appearance).

### Eyes

The position of the eyes is important. The distance between the outer canthi can be divided into equal thirds and the length between the canthus of one eye should cover the distance between the two eyes. Hypertelorism is a wide-set eye and hypotelorism is a close-set eye. Upslanting palpebral fissures mean that the outer canthus of the eye is higher than the inner canthus, while downslanting palpebral fissures are when the outer canthus is lower than the inner canthus. An example is an infant with Apert syndrome showing hypertelorism and downslanting palpebral fissures. Blepharoptosis or ptosis is the drooping of the upper eyelid below its normal position (1 mm–2 mm of upper limbus). This can be congenital, such as in the levator palpebrae muscle, or secondary to neurological injury, including third nerve palsy and Horner’s syndrome. Further assessment should be performed for other cranial nerves.

### Ears

The ear position is assessed by extending an imaginary line from the inner to the outer canthus of the eye toward the ear. The ear should be 1/3 above the imaginary line. Evidence of pits, tags, or periauricular sinus tract is assessed, as this may require further follow-up treatment. A poorly formed external ear should alert the examiner to a possible syndromic infant such as Down syndrome, whereby the ears are small with an overfolded helix. Hence, other positive clinical findings should also be assessed. Infants usually respond to the sound of their mother’s voice. The examiner may might the mother if the child turned their head to sounds or familiar voices. Subsequently, younger infants may blink or become startled by clapping or a bell.

### Skin and Spine Examination

The optimal visualisation of the skin and spine requires the infant to turn prone or side-lying. The evidence of hair tufts, dimples, or tracts along the spine is inspected, which might indicate the presence of a spinal dysraphism. A light brown to dark brown café-au-lait macules ≥ 0.5 cm in diameter or > 6, particularly in the presence of axillary freckling, requires evaluation for neurofibromatosis (NF) (29). Meanwhile, hypopigmented white skin lesions (ash leaf spots), often elliptical, are suggestive of tuberous sclerosis, a genetic disorder associated with benign multiorgan tumor growths that can impair organ functions, e.g. brain, heart, eyes and kidney (30).

### Cranial Nerve Function (27, 31–33)

General observation of the infant gives much information on cranial nerve functions. The examiners have to be opportunistic in their approach during the examination according to the infant’s temperament and leave the stressful tests for last. The olfactory nerve for smell is rarely tested. However, one can still appreciate it by testing using a strong scent, such as mint or clove, and observing for grimacing. The eye examination starts with a general inspection of the eyes to look for ptosis, squint, or stationary nystagmus. Next is the test for direct and indirect pupillary reflexes. The vision is checked by moving a bright object side to side and in an H shape 10 in–12 in from the infant’s eye. The examiner should observe for spontaneous eye movement, symmetry and nystagmus. When there is a weakness in muscle movement, the doll’s eye maneuver may be useful to differentiate the weakness of the lateral rectus (for the abducens nerve). The rooting reflex, if still present, allows appreciation of the sensory test for the trigeminal nerve. The effective sucking and swallowing of the child provide some information about the cranial nerves V, VII, IX, X, and XII. During the interaction, the examiner should notice the facial expression and response to the parent’s voice. When the baby is being fed or crying, forehead wrinkling, nasolabial fold, sucking movement and crying quality are also noted.

Testing the eighth nerve is not easy in small children, and the startle reflex can be used. Children below the age of 4 months–6 months do often not turn to sounds during the hearing distraction test since they lack attention to sounds. In many places, children are now universally screened for hearing deficit shortly after birth by otoacoustic emission (OAE) or automated auditory brain stem response (AABR) (33).

The auditory response requires the sternocleidomastoid muscle range of motion for rotation and lateral flexion, which is innervated by the spinal accessory nerve. The infant’s head must be in a neutral position, and the muscle must be palpated and compared on each side. A screening test by turning the infants’ head side to side and comparing the shoulder height is the initial evaluation in the clinic. Muscle function of the lateral flexor muscles of the neck is assessed using the muscle function scale for an infant. Further testing using an arthrodial protractor is needed when there is a lack of muscular strength or an imbalance in muscle function (34).

Some tests can be stressful and require parents to be around to keep the infant comfortable. If the infant is quiet, a fundoscopic exam is necessary. Vestibular reflex may be performed, although it is seldom included in the routine neurologic examination. The parent can assist by holding the infant and turning them around. During the test, the eye will follow the direction of rotation. Upon stopping, the eye will turn in the opposite direction. Sometimes, horizontal nystagmus is observed.

Next, an inspection of the mouth is required. The parent may have to hold the infant’s head steady. The tongue is inspected for symmetry and fasciculation using a wooden tongue blade and a good light source. The palate is checked for a cleft or a high arch by inserting the finger. Simultaneously, the sucking reflex is tested. Most of the time, the gag reflex is not routinely tested if the examiner is confident that the rest of the lower cranial nerve tests are normal, has observed the infants during bottle feeding and the parent does not report any trouble during feeding.

### Tone and Muscle Strengths

Assessment of the tone and muscle strength in infants requires good observation of the resting posture followed by assessment of the passive, active and axial tones. Diminished or lack of resistance to muscle movement or stretch indicates hypotonia, while increased muscle contractility and resistance to passive muscle movement or stretch indicate hypertonia. The primitive reflexes are useful in assessing the motor component of the limbs and trunk. In infants younger than 2 months, the palmar grasp reflex helps assess the distal power and the Moro reflex for the proximal power. As the child gets older, the upper limbs can be tested by giving a small object and watching how the infant reaches overhead and how they manipulate small objects.

Most newborn infants, either premature or full-term, have a head turn preference toward the right side (35). This is a normal asymmetry in cerebral function, with activation of the left hemisphere during the programming of the motor output, facilitating head movement to the right (27, 35). Observation of the limb position, the quality of spontaneous movements and the symmetry of movements may provide clues of possible abnormalities of the affected limbs. For example, the predominant hand position is the thumb in-fist posture intermittently loosely open spontaneously. An increase in tone may present as a ‘cortical thumb sign’ where the hand is tightly-fisted and does not open spontaneously (36). The sign is associated with an injury to corticospinal pathways.

The passive tone assesses the resistance of the appendicular muscles of the upper and lower limbs, with arm and leg tractions being commonly tested. The infant should remain supine and quiet when the movement is applied. Sometimes, clinical assessment can be difficult when the infant is stressed or crying, and interpretation may err towards hypertonia rather than a normal tone. Arm traction is when the examiner holds the infant’s wrist and attempts to pull the arm to a vertical position. The infant will flex the elbow to keep muscle resistance as the shoulder lifts off the surface. Leg traction is when the examiner holds the infant’s ankle and attempts to pull the leg to a vertical position. The infant will flex the knee and maintain this position as the buttocks lift off the surface. There will be too little resistance (extension) to traction in hypotonia.

The assessment of the active axial tone involves four tests: i) pull-to-sit (head lag), ii) head and trunk tone, iii) horizontal suspension and iv) vertical suspension.

- Pull-to-sit manoeuver (traction response) requires the baby to lie supine. The examiner grasps the infant’s wrists and slowly pulls them to a sitting position. At 2 months–3 months, an infant could activate the neck flexor muscles and hold the head in line with the trunk. The hip will flex to brace the impact of sitting. Following further encouragement, such as toys or an animated face, the infant will hold the head for a short while. However, an infant with axial hypotonia does not have anticipatory neck flexor muscle activity, and the head lags behind the trunk. Meanwhile, in axial hypertonia, the head pulls in front of the trunk (37). Asymmetric traction response may occur in an infant with monoparesis. The affected arm will be hypotonia, and the unaffected side is pulled quicker than the affected side. An example is Erb’s palsy, proximal brachial plexus injury with a predominance of the C5 and C6 nerve involvement. The incidence is between 1.6 and 2.6 in 1,000 live births (38). It is sometimes referred to as a bad shoulder with a good hand, where the arm is adducted and internally rotated with elbow extension and wrist flexion.
- Head and trunk tone. Head control is established by 3 months old of age, followed by the progressive development of trunk control (39). However, the ability is delayed in premature infants (40). The assessment of the head and trunk tone includes the ability of the infant to stabilise the head during sitting postural control. During the test, the infant will sit on the surface, facing the examiner at face level. The infant’s hands should not be touching the surface. The examiner then applies firm manual support horizontally around the trunk and observes the ability of the infant to maintain the head erect. If the infant can do so, the examiner will continue by attempting to tilt the infant forward, backward or laterally. The ability of the infant to maintain head control is observed for a minimum of 5 sec (39).
- Horizontal (ventral) suspension requires turning the infant to a prone position. The examiner will hold and support the infant at the chest and abdominal area and let the arms and legs loose. A normal infant could maintain the back straight with the head erect and in line with the back. The arms and legs are in the flexed position. Hypotonia is when the back is curved, the head flops down and the limbs hang straight (37).
- Vertical suspension requires the examiner to place both hands in the infant’s axillae and lift them upright. In a normal infant, the shoulder muscles should have sufficient strength against the examiner’s hand to allow the infant to maintain vertically with the head in line with the back. However, in hypotonia, the infant’s head will fall forward, and the infant slips through at the shoulder. During this manoeuver, the child tends to flex the legs. In children with peripheral hypertonia, fixed extension and scissoring of the lower limbs may be observed.

### Deep Tendon Reflexes

Deep tendon reflexes (DTR) are performed later in the exam to prevent the infant from getting stressed. Very premature infants of < 33 weeks old had an overall diminished reflex intensity compared to late premature infants of 33–36 weeks old (41). The reflexes develop in a caudocephalad pattern, with the lower extremities preceding those in the upper extremities and the distal reflexes preceding that of the proximal (42).

The biceps, brachioradialis and triceps reflexes are commonly tested for the upper limb, while the hip adductor, knee and ankle reflexes are for the lower limb. Tendon reflexes diminish with repeated stimulation, a phenomenon called ‘habituation’ (27). If the reflex is not visible, a finger is placed over the tendon to detect muscle contraction. DTR is based on the National Institute of Neurological Disorders and Stroke (NINDS) (43, 44). The grade is 0–4+, with grade 0 indicating no response and always abnormal, 1+ diminished, 2+ a brisk response or normal, 3+ brisker than average and 4+ when a tap elicits a repeating reflex (clonus). The 5–10 beats in infants younger than 3 months is considered normal if the clonus is symmetrical and no other concerning neurologic signs are present (45).

There are some differences when comparing DTR between infants and adults. Term neonates or infants can have brisk reflexes and still be normal. The absence of DTR is a more worrying finding than brisk reflexes. A crossed adductor reflex is a contraction of the contralateral adductors causing extension of the thigh following the tapping of the ipsilateral patella tendon. This is a normal finding in infants; as they grow, it is still present in < 10% after 8 months old (27).

### Primitive Reflexes

Primitive reflexes provide information about the development of the brainstem and cortical function. Around 4–6 months, the maturation of the CNS progresses and the primitive reflexes begin to diminish (32). The persistence beyond those times signifies CNS dysfunction (45). Palmar reflex and rooting reflexes are to disappear early, around 2 months old to 3 months old of age. The spinal Galant reflex usually integrates by 6 months. In a normal infant, a sideway flexion toward the stimulated side can be appreciated when stroking along one side of the spine. This follows by an asymmetric tonic neck reflex that disappears by 6 months old of age. An exaggerated reflex may indicate infants suffering from a static encephalopathy or some cerebral insult (46).

Capute and colleagues (47) classify the primitive reflexes in infancy into five scoring systems. Each reflex is categorised as 0 (absent), 1 (minimally present, as by tone changes), 2 (physiologically present and readily visible), 3 (more strongly present) and 4 (obligatory or controlling the patient) (4). The Moro reflex disappears around 6 months to 8 months. It is elicited by pulling the infant halfway to a sitting position from supine and letting the head fall back. The reflex is produced by the suddenness of this stimulus, not the distance of the fall. The sudden movement causes a rapid abduction and extension of arms and opening of hands. The back muscle starts to tense, the hip will flex, and often the infant starts to cry.

Plantar grasp disappears by 7 months to 9 months to allow the infant to start standing. The stepping reflex is provoked by holding the infant under the axilla in an upright position and placing the anterior tibia or dorsum of the infant’s foot under or against the edge of a table. The infant’s hip and knee will flex, simulating walking. The response diminishes by the end of the first year (32). Asymmetry may indicate a lesion in the basal ganglia, brainstem, or spinal cord (46). Lastly, the sucking reflex should integrate by 10 months to 12 months (27).

The above examination may not be assessed in detail, particularly by other clinicians outside the pediatric clinical setting; hence, Video 3 (https://youtu.be/CkNHxnhI8PY) serves as a general summary that one should look for in the clinic setting.

### Sensory Examination

The Achilles heel of sensory examination is unreliable responses. Hence, it is not as thorough as in adults and is limited to evaluating response to touch and, if required, to pain. Evaluation of the neonate’s sensory system involves touching the face and stroking different dermatomes of the trunk and limbs with a cotton applicator or sharper objects (clip, broken tongue applicator) (31). Typically, the infant may look at the area being stimulated. The examiner should observe for the facial response (such as grimacing) or change in behavior after each maneuver (32). In babies with paralysis, the pain can be seen from facial expressions and failure to withdraw from the pain. Assessment of the sensation according to ascending dermatomes may be useful in patients with spina bifida with myelomeningocele.

## Summary

The neurological examination techniques for neonates to infants require skills and a lot of patience and practice. Observing the infants alone does give many clues, nevertheless, perfecting the examination is a requisite. Modification is needed to correspond to different developmental stages and one cannot avoid examining temperamental infants. Hopefully, this article and videos will be useful to the students and other clinicians in helping them with their decision-making.

## Figures and Tables

**Table 1:** Observation of level of alertness

Level of alertness	Description
Normal	Normal respond to arousal with normal motor movements of the limbs and face
Slight to moderate stupor	Sleepy with diminished or absent arousal and motor responses
Deep stupor or coma	Cannot be aroused; motor response are diminished or absent (coma)
Irritable	Agitated and cannot be soothed; cries with minimal stimulation
Lethargic	Unable to maintain an alert state
